# The effectiveness of integrative medicine interventions on pain and anxiety in cardiovascular inpatients: a practice-based research evaluation

**DOI:** 10.1186/1472-6882-14-486

**Published:** 2014-12-13

**Authors:** Jill R Johnson, Daniel J Crespin, Kristen H Griffin, Michael D Finch, Rachael L Rivard, Courtney J Baechler, Jeffery A Dusek

**Affiliations:** Penny George Institute for Health and Healing, Allina Health, 800 East 28th Street, MR 33540, Minneapolis, MN 55407-3799 USA; Division of Health Policy and Management, University of Minnesota School of Public Health, 420 Delaware Street SE, MMC 729, Minneapolis, MN 55455 USA; Finch and King, Inc, 5917 Girard Ave South, Minneapolis, MN 55419 USA

**Keywords:** Integrative medicine, Cardiology, Pain, Anxiety, Inpatients, Complementary medicine

## Abstract

**Background:**

Pain and anxiety occurring from cardiovascular disease are associated with long-term health risks. Integrative medicine (IM) therapies reduce pain and anxiety in small samples of hospitalized cardiovascular patients within randomized controlled trials; however, practice-based effectiveness research has been limited. The goal of the study is to evaluate the effectiveness of IM interventions (i.e., bodywork, mind-body and energy therapies, and traditional Chinese medicine) on pain and anxiety measures across a cardiovascular population.

**Methods:**

Retrospective data obtained from medical records identified patients with a cardiovascular ICD-9 code admitted to a large Midwestern hospital between 7/1/2009 and 12/31/2012. Outcomes were changes in patient-reported pain and anxiety, rated before and after IM treatments based on a numeric scale (0-10).

**Results:**

Of 57,295 hospital cardiovascular admissions, 6,589 (11.5%) included IM. After receiving IM therapy, patients averaged a 46.5% (p-value < 0.001) decrease in pain and a 54.8% (p-value < 0.001) decrease in anxiety. There was no difference between treatment modalities on pain reduction; however, mind-body and energy therapies (p-value < 0.01), traditional Chinese medicine (p-value < 0.05), and combination therapies (p-value < 0.01) were more effective at reducing anxiety than bodywork therapies. Each additional year of age reduced the odds of receiving any IM therapy by two percent (OR: 0.98, p-value < 0.01) and females had 96% (OR: 1.96, p-value < 0.01) higher odds of receiving any IM therapy compared to males.

**Conclusions:**

Cardiovascular inpatients reported statistically significant decreases in pain and anxiety following care with adjunctive IM interventions. This study underscores the potential for future practice-based research to investigate the best approach for incorporating these therapies into an acute care setting such that IM therapies are most appropriately provided to patient populations.

## Background

Pain and anxiety occurring from acute care for cardiovascular disease (CVD) are associated with long-term health risks. Specifically, preoperative anxiety and acute postoperative non-anginal pain in cardiac patients have been found to predict persistent postoperative pain up to 24 months after surgery [1]. Furthermore, anxiety shortly after hospitalization for myocardial infarction has been reported as a strong predictor of recurrent myocardial infarction [2]. Given that pain and anxiety symptoms add to the physical and psychosocial burden of post-operative CVD patients, it is important to find effective approaches to ameliorate these symptoms.

Pharmacological symptom management among cardiovascular patients, while often appropriate and necessary, presents challenges such as side effects, limited effectiveness, and risk of adverse events [3]. In 2005, the American College of Cardiology Foundation Task Force recommended consideration of non-pharmacological options for cardiovascular patients, including the use of complementary and alternative medicine (CAM) therapies [4]. A 2012 systematic review of hospitals and outpatient clinics reported that between four and sixty-one percent of cardiovascular patients use CAM for managing cardiovascular conditions and maintaining general health [5]. Integrative medicine (IM) includes the use of CAM therapies, such as massage, acupuncture, and mind-body therapies, in conjunction with conventional medicine.

Several systematic reviews report on efficacy of IM for CVD-related outcomes and risk factors including blood pressure [6–9], blood lipids and cholesterol [9], and psychosocial outcomes [9] in outpatient settings. Although there is some evidence for efficacy, conclusions are limited by methodological challenges [4, 6–9].

For inpatients with cardiovascular diagnoses, randomized controlled trials specifically focused on the use of IM therapies for symptom management have reported improvements in pain and anxiety, among other outcomes [10–17]. Trials are important for testing the efficacy of these therapies; however, it has been suggested that the evidence hierarchy that prioritizes RCTs over other methods may warrant revising for the examination of certain interventions [18]. Real-world observational data is critical for better understanding the effectiveness of integrative therapies for cardiovascular inpatients [19], and observational methods may be particularly appropriate for studying IM, despite the inherent limitation of not being experimental [20]. Practice-based research is also important for improving clinical practice by delivering recommended care in the most appropriate manner for specific populations [18, 21].

In 2010, our research group reported a 55.8% average reduction in pain with IM use across 1,837 inpatients at Abbott Northwestern Hospital (ANW), based on a retrospective medical record review; however, results for cardiovascular patients were not separately analyzed at that time [22]. In the current study, we evaluate the effectiveness of IM therapy, as an adjunct to standard care, for treating pain and anxiety, focusing on a large, inpatient cardiovascular population at ANW. To our knowledge, this is the first study in which multiple IM therapies are studied using practice-based research methodology among cardiovascular inpatients to treat pain and anxiety.

## Methods

### Study design and setting

This retrospective, practice-based research study of cardiovascular inpatients was conducted at ANW, a 630-bed teaching and specialty hospital in Minneapolis, MN. The Penny George Institute for Health and Healing (PGIHH) at ANW was founded in 2003 and offers hospitalized patients, through electronic physician and nurse referrals, a wide-array of integrative health services at no charge to patients [23]. All IM practitioners at PGIHH are formally trained and have necessary licensure and/or certification in their area of specialty (e.g., aromatherapy, acupuncture, massage, music). Referral criteria include: (a) the patient is able to participate in integrative health intervention, and (b) patient concerns include pain, anxiety/stress, elimination problems, nausea/vomiting, insomnia, coping with change in health/well-being, or maintaining/prolonging a pregnancy.

### Study population

All cardiovascular inpatients age 18 years or older at ANW, who were admitted between July 1, 2009 and December 31, 2012, were included in the study population. Patients seen as outpatients, in the emergency room, and who were in the hospital solely for observation were excluded. Medical record data were obtained on all eligible inpatients and cardiovascular patients were retrospectively identified. All patients whose medical record data were obtained gave written permission upon hospital admission to use their records for research purposes.

The study population included those with diseases of the circulatory system, identified using the International Classification of Diseases (ICD), 9^th^ Revision, Clinical Modification diagnosis codes (390-459). Any admission that had at least one of these ICD-9 codes as the admission’s primary or secondary diagnosis or any hospital encounter-level diagnosis was eligible for the study.

We created non-mutually exclusive indicators pertaining to five circulatory system diseases: diseases of arteries, arterioles and capillaries (440-448); cerebrovascular disease (430-438); hypertensive disease (401-405); ischemic heart disease (410-414); and diseases of pulmonary circulation (415-417). Patients of all other circulatory system diseases were grouped into an ‘other’ category.

The study was approved by the Institutional Review Board of Allina Health with a waiver of informed consent.

### Measurements

#### Demographic and admission characteristics

Data extracted from medical records included patient age at time of admission, sex, race, marital status, and health insurance status. The data included the All Patient Refined Diagnostic Related Groups (APR-DRG) [24] severity of illness measures calculated from patients’ diagnoses codes. The measure includes four categories of severity: 1) minor, 2) moderate, 3) major, and 4) extreme. Data pertaining to each IM session were routinely documented in a customized documentation flowsheet within the medical records.

#### Integrative medicine therapies

IM practitioners used their clinical judgment to provide therapies, within their scope of practice, they deemed necessary and therapeutic for each patient, after consulting with the patient. Many patients received IM therapy numerous times throughout their hospital admission. The term ‘session’ is used to define each unique administration of IM therapy, distinguished by time of procedure, within a hospital admission. For the present analyses, IM therapies were placed into one of three broad categories: bodywork (BW), which included craniosacral therapy, medical massage, and reflexology; mind-body and energy therapies (MBE), which was divided into separate mind-body and energy subcategories; and traditional Chinese medicine (TCM), which included acupressure, acupuncture, and Korean hand therapy. Importantly, patients could receive therapy from more than one category during each session, which has been defined as combination therapies. The presence or absence of each of these IM therapies was coded at each session such that BW, MBE, TCM, and any combination of these therapies were mutually exclusive.

#### Pain and anxiety scores

IM practitioners collected patients’ self-reported pain and anxiety scores directly prior to and after each IM session. Practitioners requested patients to provide a single number to indicate the level of pain they were currently experiencing on an 11-point numeric rating scale where 0 was defined as ‘no pain’ and 10 was defined as ‘worst pain imaginable’. Similarly, practitioners recorded anxiety scores using the same methodology, where 0 was ‘no anxiety’ and 10 was ‘worst anxiety imaginable’. The primary endpoints were changes in pain and anxiety scores, calculated by subtracting the pre-score from the post-score. Zero to 10 numeric rating scales for pain have been found valid and reliable [25, 26].

### Analytic data set

A total of 57,444 cardiology-related hospital admissions were identified from medical records. During data cleaning, 149 hospital admissions were removed due to missing demographic data (51 admissions) or inability to determine severity of illness (98 admissions), resulting in 57,295 cardiology admissions from 37,259 unique patients. Of the 57,295 admissions, 6,589 (11.5%) had 16,344 IM therapy sessions (average of 2.48 per admission). In many cases, practitioners were unable to collect pre- or post-pain and anxiety scores or patients reported no pain or anxiety. Only patients who reported pre- and post-pain scores and/or pre- and post-anxiety scores, and pre-pain/pre-anxiety scores greater than zero, were included in the subsequent analyses examining changes in pain and anxiety after receiving IM therapy.

Because IM therapies were observed at the hospital admission level, but pain and anxiety scores were assessed at the IM session level, one session was randomly selected from each remaining hospital admission in order to keep the level of analysis consistent between the selection and score change equations (see below). Thus, we dropped all hospital admissions with only missing scores or pre-pain or -anxiety scores equal to zero. This method produced a sample of 54,163 hospital admissions for the pain model, of which 3,457 (6%) had IM therapy, and 52,572 admissions for the anxiety model, of which 1,866 (4%) had IM therapy.

### Statistical analysis

#### IM therapy utilization

Logistic regression was used to predict the probability of receiving any IM therapy during a hospital admission as a function of patient demographics, circulatory system disease diagnosis, severity, and health insurance status, and odds ratios for each covariate are presented. A p-value of less than 0.05 was used to signify statistical significance. We used a random sample of 25,000 observations to test the goodness-of-fit for our model using the Hosmer-Lemeshow test [27]. We did not use the full sample because the Hosmer-Lemeshow test has been shown to likely reject the null hypothesis of a good fit even for models that fit well when the sample size is greater than 25,000 due to increased statistical power [28]. The percent of admissions correctly classified by the model were also calculated.

#### Pain and anxiety

First, to determine if IM therapies were associated with reductions in pain and anxiety, paired t-tests were conducted using the null hypothesis that the pre- and post-pain or anxiety scores were equal.

Next, multivariate regression was used to estimate reductions in pain and anxiety during IM sessions. Because patients receiving IM therapy may systematically differ from the general sample of cardiovascular patients, an ordinary least squares model could produce bias parameters when generalizing results. To address this bias, a Heckman selection model [29] was used to account for selection into the sample of IM therapy recipients.

To correctly identify the parameters that affect pain and anxiety, at least one variable in the selection-equation (i.e. utilization of IM therapy) should be specified which predicts IM therapy use, but does not affect changes in pain or anxiety. Since marital status and health insurance status were expected to fit this criterion, our model predicted selection into the sample of IM sessions using all patient demographic, circulatory system disease diagnosis, severity, and health insurance variables (the same set of covariates as our logistic regression predicting IM therapy use). Changes in pain and anxiety scores were estimated using diagnosis, age, sex, race, severity, and the inverse Mills ratio calculated from the selection-equation to control for selection. Additionally, we estimated a second model, which included IM therapy categories, to determine if differential effects between the categories existed.

All analyses were conducted in Stata Version 13 (StataCorp LP; College Station, TX).

## Results

### Descriptive statistics

Of the 57,295 hospital admissions over the study period, 6,589 (11.5%) included IM therapy (Table 1). The mean age of inpatients utilizing IM therapies (63.5 years) was approximately four years younger than inpatients not receiving IM therapies (67.8 years). Fifteen percent of women received IM therapy compared to only 9% of men. IM hospital admissions were comprised of patients with significantly higher illness severity. A total of 16,344 IM therapy sessions were administered for an average of 2.48 sessions per hospital admission (Table 2). BW comprised 45.7% of IM sessions compared to 12.6% for MBE, 18.7% for TCM, and 23.0% for combination therapies.

**Table 1:** **Abbott Northwestern cardiovascular inpatient characteristics (n = 57,295)** ^**a**^

	No IM Therapy	IM Therapy	p-value ^b^
	(n = 50,706)	(n = 6,589)	
*Age (years ± SD)*	67.8 ± 15.4	63.5 ± 14.8	<0.001
*Sex (%)*			
Female	23,649 (46.6)	4,076 (61.9)	<0.001
Male	27,057 (53.4)	2,513 (38.1)	<0.001
*Race (%)*			
White	45,285 (89.3)	6,058 (91.9)	<0.001
African American	3,550 (7.0)	327 (5.0)	<0.001
Asian	945 (1.9)	106 (1.6)	0.147
Other	926 (1.8)	98 (1.5)	0.051
*Marital Status (%)*			
Married	26,475 (52.2)	3,572 (54.2)	0.002
Widow	9,821 (19.4)	977 (14.8)	<0.001
Divorced	4,865 (9.6)	723 (11.0)	<0.001
Single	9,545 (18.8)	1,317 (20.0)	0.023
*Circulatory System Disease (%)*			
Arteries, Arterioles & Capillaries	4,379 (8.6)	427 (6.5)	<0.001
Cerebrovascular	5,994 (11.8)	863 (13.1)	0.003
Hypertension	28,556 (56.3)	4,207 (63.8)	<0.001
Ischemic Heart Disease	15,016 (29.6)	1,278 (19.4)	<0.001
Pulmonary Circulation	2,408 (4.7)	481 (7.3)	<0.001
Other	23,485 (46.3)	3,365 (51.1)	<0.001
*APR-DRG Severity (%)*			
Minor	7,926 (15.6)	497 (7.5)	<0.001
Moderate	19,179 (37.8)	1,928 (29.3)	<0.001
Major	18,049 (35.6)	2,745 (41.7)	<0.001
Extreme	5,552 (10.9)	1,419 (21.5)	<0.001
*Health Insurance (%)*			
Commercial	15,084 (29.7)	2,368 (35.9)	<0.001
Medicare	26,492 (52.2)	3,045 (46.2)	<0.001
Medicaid	7,951 (15.7)	1,009 (15.3)	0.440
Other	1,179 (2.3)	167 (2.5)	0.291

^a^Total n refers to number of hospital admissions and not individual patients.

^b^P-value calculated from two-tailed t-test comparing no IM therapy and IM therapy, adjusted for equal or unequal variances as appropriate.

**Table 2:** **Distribution of IM sessions by treatment type and circulatory system disease**

	Diseases of the circulatory system						
	Any ^a^	Arteries, Arterioles & Capillaries	Cerebrovascular	Hypertension	Ischemic heart disease	Pulmonary circulation	Other
No. of Cardiovascular Admissions with IM Therapy	6,589	427	863	4,207	1,278	481	3,365
No. of Total IM Sessions (%)	16,344 (100.0)	1,304 (100.0)	2,798 (100.0)	9,868 (100.0)	2,862 (100.0)	1,776 (100.0)	9,216 (100.0)
Bodywork (BW) (%)	7,477 (45.7)	638 (48.9)	1,479 (52.9)	4,455 (45.1)	1,359 (47.5)	947 (53.3)	4,554 (49.4)
Mind-Body and Energy Therapies (MBE) (%)	2,059 (12.6)	168 (12.9)	311 (11.1)	1,108 (11.2)	371 (13.0)	238 (13.4)	1,197 (13.0)
Traditional Chinese Medicine (TCM) (%)	3,051 (18.7)	176 (13.5)	482 (17.2)	2,108 (21.4)	398 (13.9)	186 (10.5)	1,367 (14.8)
Combination Therapy (%)	3,757 (23.0)	322 (24.7)	526 (18.8)	2,197 (22.3)	734 (25.6)	405 (22.8)	2,098 (22.8)

^a^IM sessions for patients with more than one circulatory system disease during an admission count under the ‘Any’ column as well as under the appropriate disease columns. As such, the sum of individual disease columns does not equal the sum of the ‘Any’ column.

### IM therapy utilization analysis

Similar to our descriptive statistics, older patients had reduced odds of receiving any IM therapy in our logistic regression model (Table 3), as each additional year of age decreased the odds of any IM therapy use by 2% (odds ratio (OR): 0.98, p-value < 0.01). Females had 96% (OR: 1.96, p-value < 0.01) higher odds of receiving any IM therapy during a hospital admission compared to males. Compared to other diseases of the circulatory system, admissions from hypertensive disease (OR: 1.48, p-value < 0.01), and diseases of pulmonary circulation (OR: 1.23, p-value < 0.01) were associated with increased odds of receiving IM therapy, while ischemic heart disease (OR: 0.73, p-value < 0.01) and diseases of arteries, arterioles, and capillaries (OR: 0.75, p-value < 0.01) had reduced odds of receiving IM therapy.

**Table 3:** **Odds ratio for IM use among cardiovascular inpatients** ^**a**^

	OR	SE
*Age (years)*	0.98^b^	0.00
*Sex (Reference = Male)*		
Female	1.96^b^	0.06
*Race (Reference = White)*		
African American	0.47^b^	0.03
Asian	0.57^b^	0.08
Other/Unknown	0.63^b^	0.07
*Marital Status (Reference = Married)*		
Widowed	0.73^b^	0.04
Divorced	0.91	0.05
Single	0.79^b^	0.03
*Circulatory System Disease (Reference = Other)*		
Arteries, Arterioles & Capillaries	0.75^b^	0.04
Cerebrovascular	1.08	0.05
Hypertension	1.48^b^	0.04
Ischemic Heart Disease	0.73^b^	0.03
Pulmonary Circulation	1.23^b^	0.07
*Severity of Illness (Reference = Minor)*		
Moderate	1.68^b^	0.09
Major	2.85^b^	0.15
Extreme	5.09^b^	0.30
*Health Insurance (Reference = Commercial)*		
Medicare	0.88^b^	0.03
Medicaid	0.86^b^	0.04
Other	0.94	0.09

^a^Odds ratios from logistic regression of 6,589 cardiology admissions. The dependent variable was administration of IM therapy during admission. OR indicates odds ratio; SE, standard error.

^b^p < 0.01.

The p-value from a Hosmer-Lemeshow test was 0.58, indicating a good fit. The model correctly classified 88% of hospital admissions as receiving IM or not receiving IM. Although this result was driven by the model’s under-prediction of IM hospital admissions and the large proportion of non-IM hospital admissions, there was a significant difference (p-value < 0.001) in the predicted probability of receiving IM therapy between the IM hospital admissions group (predicted probability = 0.16) and the non-IM hospital admissions group (predicted probability = 0.11).

### Pain and anxiety analysis

Sessions with IM therapy resulted in, on average, a 46.5% (p-value < 0.001) decrease in pain score (Table 4). Anxiety scores decreased by an average of 54.8% (p-value < 0.001) after the administration of IM therapies (Table 4).

**Table 4:** **Mean pre- and post-IM therapy pain and anxiety scores by circulatory system disease and therapy type** ^**a**^

Circulatory system disease		Any	Arteries, Arterioles & Capillaries	Cerebrovascular	Hypertension	Ischemic heart disease	Pulmonary circulation	Other
Any treatment	No. Pain Obs	5,981	454	713	3,702	1,122	574	3,180
	% Decrease in Pain	46.5	46.8	49.5	45.2	47.6	49.2	48.2
	SE	0.5	1.7	1.4	0.6	1.1	1.5	0.6
	No. Anxiety Obs	3,109	227	293	1,663	696	370	1,871
	% Decrease in Anxiety	54.8	53.1	55.0	53.2	53.0	52.9	55.2
	SE	0.6	2.2	1.8	0.8	1.2	1.6	0.7
BW	No. Pain Obs	2,761	212	395	1,644	532	303	1,580
	% Decrease in Pain	46.6	45.4	50.7	44.9	48.1	49.6	48.6
	SE	0.6	2.4	1.7	0.8	1.5	2.0	0.8
	No. Anxiety Obs	1,454	103	155	779	305	195	900
	% Decrease in Anxiety	51.7	48.1	51.5	50.0	49.0	52.0	53.1
	SE	0.8	3.2	2.4	1.1	1.8	2.1	1.0
MBE	No. Pain Obs	318	26	25	173	49	32	173
	% Decrease in Pain	41.8	54.6	44.0	41.2	41.3	43.9	42.3
	SE	2.3	8.2	7.8	2.9	8.8	6.6	2.5
	No. Anxiety Obs	290	16	28	141	57	25	167
	% Decrease in Anxiety	57.0	67.3	62.7	55.3	59.8	55.7	56.0
	SE	1.8	7.0	4.2	2.6	4.5	6.1	2.3
TCM	No. Pain Obs	1,248	63	122	932	154	64	490
	% Decrease in Pain	48.4	46.3	53.0	47.1	44.6	59.7	51.9
	SE	1.3	4.9	3.6	1.5	3.6	4.9	1.8
	No. Anxiety Obs	200	8	15	120	22	15	105
	% Decrease in Anxiety	71.5	72.9	88.7	68.5	72.5	69.2	67.3
	SE	2.6	10.5	5.2	3.8	6.7	8.4	4.0
Combination	No. Pain Obs	1,654	153	171	953	387	175	937
	% Decrease in Pain	45.7	47.6	45.0	44.4	48.8	45.5	46.8
	SE	0.9	2.8	3.1	1.1	1.9	2.6	1.2
	No. Anxiety Obs	1,165	100	95	623	312	135	699
	% Decrease in Anxiety	55.3	54.3	53.2	53.8	54.2	51.8	55.9
	SE	0.9	3.3	3.3	1.3	1.8	2.7	1.2

^a^BW indicates bodywork; MBE, mind-body and energy therapies; TCM, traditional Chinese medicine; SE, standard error. All results statistically significant (p < 0.001).

A Heckman selection model was used to account for selection into the sample of IM therapy recipients. Results from this model predict that for a male with mean age (67.3), mean inverse Mills ratio (2.05), and the modal value of all categorical variables (i.e. White, hypertension diagnosis, and moderate severity), IM therapy was associated with a 1.81 (p-value < 0.001) point reduction in pain (calculated from coefficients shown in Base Model; Table 5). This result represents a 36.2% (p-value < 0.001) reduction in pain for a male with the mean pain pre-score (5.00). For a female with the same admission attributes, IM therapy was associated with a 40.6% (p-value < 0.001) reduction in pain. When IM therapy categories were included in the regression analysis, we found no significant difference by IM therapy type. The inverse Mills ratio had an insignificant effect on pain, suggesting that selection bias was not present.

**Table 5:** **Predicted change in pain and anxiety scores** ^**a**^

		Base model		Model including treatment types	
Outcome		Marginal effect	SE	Marginal effect	SE
Pain					
	*Age*	0.00	0.01	0.00	0.01
	*Female*	-0.23	0.13	-0.23	0.13
	*Race (Reference = White)*				
	African American	-0.35	0.21	-0.35	0.21
	Asian	-0.35	0.25	-0.34	0.25
	Other	0.25	0.29	0.24	0.29
	*Circulatory System Disease (Reference = Other)*				
	Arteries, Arterioles & Capillaries	-0.10	0.12	-0.11	0.12
	Cerebrovascular	0.07	0.11	0.07	0.11
	Hypertension	0.06	0.10	0.06	0.10
	Ischemic Heart Disease	-0.05	0.10	-0.05	0.10
	Pulmonary Circulation	0.17	0.13	0.17	0.13
	*Severity (Reference = Minor)*				
	Moderate	-0.24	0.14	-0.24	0.14
	Major	-0.42^b^	0.19	-0.43^b^	0.19
	Extreme	-0.45	0.27	-0.46	0.28
	*Treatment Type (Reference = BW)*				
	MBE	.	.	0.22	0.13
	TCM	.	.	-0.02	0.08
	Combination	.	.	-0.03	0.07
	*Inverse Mills Ratio*	-0.21	0.43	-0.22	0.43
	*Constant*	-1.22	0.72	-1.23	0.72
Anxiety					
	*Age*	0.03^b^	0.01	0.03^b^	0.01
	*Female*	-0.56^b^	0.28	-0.62^b^	0.28
	*Race(Reference = White)*				
	African American	-0.06	0.36	-0.02	0.36
	Asian	-0.12	0.37	-0.10	0.38
	Other	0.86	0.52	0.95	0.53
	*Circulatory System Disease (Reference = Other)*				
	Arteries, Arterioles & Capillaries	-0.08	0.18	-0.05	0.18
	Cerebrovascular	0.27	0.19	0.27	0.19
	Hypertension	0.02	0.11	0.01	0.11
	Ischemic Heart Disease	-0.04	0.11	0.00	0.12
	Pulmonary Circulation	-0.05	0.20	-0.08	0.20
	*Severity (Reference = Minor)*				
	Moderate	-0.43	0.27	-0.50	0.27
	Major	-0.89^b^	0.44	-1.08^b^	0.45
	Extreme	-0.91	0.66	-1.19	0.66
	*Treatment Type (Reference = BW)*				
	MBE	.	*.*	-0.67^c^	0.16
	TCM	.	*.*	-0.42^b^	0.20
	Combination	.	*.*	-0.34^c^	0.10
	*Inverse Mills Ratio*	-1.04	0.76	-1.26	0.77
	*Constant*	-1.12	1.41	-0.38	1.42

^a^Marginal effect of covariates on the change in pain and anxiety scores after administration of IM therapy from a Heckman selection model. Marital status and health insurance were used as exclusion restrictions in the selection-equation. Hospital admissions for which all change in pain or anxiety score were missing were excluded from this analysis. The pain sample consisted of 54,163 admissions, of whom 3,457 utilized IM therapies; the anxiety sample consisted of 52,572 admissions, of whom 1,866 utilized IM therapies. BW indicates bodywork; MBE, mind-body and energy therapies; TCM, traditional Chinese medicine.

^b^p < 0.05.

^c^p < 0.01.

The Heckman selection model predicted a 2.28 (p-value < 0.001) point decrease (Table 5) or a 41.6% (p-value < 0.001) reduction in anxiety score for a male with mean age (67.3), mean inverse Mills ratio (2.36), and the modal value of all categorical variables with the mean anxiety pre-score (5.48). For females, IM therapy was associated with a 51.6% (p-value < 0.001) reduction in anxiety. At the mean pre-anxiety score, MBE (12.2 percentage points), TCM (7.7 percentage points), and combination (6.2 percentage points) therapies were all more effective than BW therapies. Additionally, MBE therapy was 6.0 percentage points more effective than combination therapy. The inverse Mills ratio had an insignificant effect on anxiety, suggesting that selection bias was not present.

## Discussion

This practice-based research study, using routinely-collected electronic medical record data, assessed the effectiveness of adjunctive IM therapy on pain and anxiety among cardiovascular inpatients. Of 57,295 hospital admissions over the study period, 6,589 (11.5%) included IM therapy. Older patients had reduced odds of receiving any IM therapy and females had 96% higher odds of receiving any IM therapy compared to males. After receiving IM therapy, patients averaged a 46.5% reduction in pain and a 54.8% reduction in anxiety. For acute pain measured by a 0-10 numeric rating scale, a clinically significant reduction in pain is 20% for minimal noticeable improvement by patients, and between 35% and 44% reduction for a more substantial improvement (for patients with moderate or severe baseline pain, respectively) [30]; therefore, our results may be interpreted as clinically significant as well as statistically significant. The changes in pain and anxiety we observed may be in addition to or concurrent with changes from analgesics and psychoactive medications or other medical therapies. This study found no difference between treatment modalities on pain reduction; however, MBE, TCM, and combination therapies were all more effective at reducing anxiety than BW therapies.

Hospital-based IM is an emerging field encompassing a wide range of practice models and arrays of services offered, and both RCTs and observational studies comprise a relatively small portion of the evidence base in the IM field [31]. The present results are generally consistent with previous reports of CAM for cardiovascular inpatients, although research to date among cardiovascular inpatient populations focuses only on bodywork and mind-body and energy therapies, not traditional Chinese medicine/acupuncture modalities. Several randomized trials involving preoperative [10] and postoperative [13–15] cardiovascular inpatients compared massage therapy to standard care with relaxation or rest and reported significant reductions in pain and anxiety scores on a 0-10 scale. A 2006 report of a randomized trial of open heart surgery patients described an unquantified reduction in pre- and postoperative pain in the intervention arm (a package of CAM therapies including guided imagery and light massage) compared with the standard care control [16]. A randomized trial among cardiac inpatients treated with healing touch found a statistically significant 6.3 point reduction in anxiety as measured by the State Trait Anxiety Inventory (STAI) [11]. One randomized trial reported statistically non-significant changes in pain and anxiety among post-surgical cardiac patients receiving foot massage and guided relaxation compared to controls; however, although not significant, the authors reported a trend indicating the interventions appeared to be effective [17]. Another randomized trial of massage reported non-significant changes in pain and anxiety in the intervention group [32], but as noted by Braun and colleagues, the standard massage sequence used in that study may have limited the potential efficacy [15].

The present study is unique for its practice-based observational design and large sample of cardiovascular inpatients. Prior investigations have been randomized trials with smaller sample sizes and more limited scope [10, 11, 13–17, 32]. Observational data are important for understanding real-world clinical effectiveness [19, 21], and appropriately analyzed observational data have been found to complement the results of randomized trials of cardiovascular outcomes [33]. Given the high levels of CAM or IM usage by cardiovascular patients [5], practice-based observational research on IM is important for the evidence base on management of CVD symptoms and recovery. An important strength of this study is our use of a Heckman selection model to adjust for any non-random selection of whether patients received IM therapy. As a result of this adjustment, our results are generalizable to cardiovascular patients at ANW. These results, however, may not generalize to other hospital settings.

This study is not without limitations. First, the present results reflect only short-term changes in pain and anxiety. Because clinical relevance may be limited with a focus on short-term changes, further investigation into the long-term effects of IM on pain and anxiety should be considered. Second, the IM practitioner who delivered the IM therapy was also responsible for collection of the pre- and post-IM therapy pain and anxiety scores. As a result, the potential exists for social desirability or other response or reporting bias. However, provider-collected scores are reflective of how pain and other symptoms are assessed in a real-world acute care setting. Physicians, nurses, and other care providers regularly ask patients to self-report their rating of symptoms, and commonly used measures such as the numeric rating scale have been reported on [25, 26]. It has been recommended that clinical decisions be based on information collected in this way [34]. Third, the effects of IM on pain scores are not separated from the pain reduction impact of pain medications. Our findings only reflect changes in pain and anxiety when IM therapies are provided adjunctively to standard medical care, which may include use of analgesics and psychoactive medications. However, because pain and anxiety are often only partially resolved by medications [35–37], investigating the use of IM therapies in conjunction with standard medical care is an important next step in learning how to better manage pain and anxiety in an acute care setting. Future practice-based research should explore the interactive effects of IM therapy and pain medication by, for example, including the timing of medication use in relation to provision of the IM therapy. Finally, this study did not explore the potential biological mechanisms of IM underlying the observed pain and anxiety reduction; this area of exploration is additionally warranted in future practice-based research.

## Conclusions

This study provided a unique opportunity to describe and investigate the effectiveness of delivering IM therapy to cardiovascular inpatients. Our results suggest that after receiving adjunctive IM therapies, cardiovascular inpatients reported statistically significant decreases in pain and anxiety. This study lays preliminary groundwork for future practice-based research to investigate the best approach for incorporating these therapies into an acute care setting such that IM therapies are most appropriately provided to patient populations.

## Abbreviations

CVD: Cardiovascular disease
CAM: Complementary and alternative medicine
IM: Integrative medicine
ANW: Abbott Northwestern Hospital
PGIHH: Penny George Institute for Health and Healing
ICD: International Classification of Diseases
APR-DRG: All Patient Refined Diagnostic Related Groups
BW: Bodywork
MBE: Mind-body and energy therapies
TCM: Traditional Chinese medicine.
